# The Influence of God and Providence on Happiness and the Quality of Life of Patients Benefiting from Aesthetic Medicine Treatments in Poland

**DOI:** 10.1007/s10943-015-0036-3

**Published:** 2015-03-31

**Authors:** Anna Galęba, Beata Bajurna

**Affiliations:** 1 Department of Social Medicine, University of Medical Sciences in Poznan, Poznan, Poland; 2 Private Practice of Aesthetic Medicine and Anti-Aging in Warszawa and Poznan, Warsaw, Poland; 3 Leonida Teligi 6 Str., 62-510 Konin, Poland; 4 5C Rokietnicka Str., 60-806 Poznan, Poland

**Keywords:** Aesthetic medicine, Cosmetic medicine, Quality of life, Perception of happiness, God, God’s Providence

## Abstract

The research reveals the impact of a belief in god and god’s Providence on the happiness and quality of life of patients benefiting from aesthetic medicine treatments in Poland (country where over 90 % of society declare to be deeply devout). The work also examines age and sex of the patients benefiting from beauty treatments (botulinum toxin, fillers, medical peels and needle mesotherapy), their quality of life and also the impact of various factors, including God and Divine Providence on their happiness. The research shows the analysis of factors influencing the successes or failures in the past year and presents the comparison of patients who have benefited from the aesthetic medicine treatments (cosmetic medicine) to the common average Polish citizens.

## Introduction

The concept of God is not an innate one, but it is gradually formed in the psyche of a humankind. It is usually being shaped from an early childhood in the process of socialization, and it is modified on the basis of one’s own cognitive and emotional experiences, primarily associated with the structure of one’s family life. Providence is in as god’s interest in everything that He has created especially a man, leading him to the full perfection and to the ultimate goal (Zuberbier 1989).

There is no specific, biblical prohibition of changing the appearance of our face, our bodies. The belief is that God gives us the freedom of choice, and our conscience must lead us and guide through the behaviours that are not explicitly prohibited for us. For this reason, one cannot find any justification for believing that aesthetic medicine treatments (cosmetic medicine) are clearly immoral; however, one must be careful not to overuse them. People are worthy, as they are made in the image of God. A person’s worth does not depend on and does not change with changes in the appearance of one’s face or body because *I have rejected him…,because man sees the appearance but the Lord looks into the heart* (1 Sm 16. 7). It is not our appearance that makes us worthy, but a belief in God whose image we have been made. Regardless of how one looks, this should not be forgotten (Poupard 2010).

The following work reveals what kind of influence of a belief in God and God’s Providence has on the happiness and quality of life of patients benefiting from aesthetic medicine treatments in Poland (country where over 90 % of society declare to be deeply devout). The work examines age and sex of the patients benefiting from beauty treatments, their quality of life and also the impact of various factors, a belief in God on their happiness.

The research shows the analysis of factors influencing the successes or failures in the past year and compares the patients who have benefited from the aesthetic medicine treatments to the common average Polish citizens.

## Materials and Methods

In the research, 603 respondents of both sexes took part; they were aged from 21 to 61 years, benefiting from the cosmetic treatments in aesthetic medicine clinics in Poland (botulinum toxin, fillers, medical peels, needle mesotherapy), randomly selected to take part in the test.

Scheffeg’s and Bonferroni’s methods and expert system (Domański and Parys 2007) were used for multiple comparisons. In the social studies, an assessment fraction error (½ confidence interval) is possible to be below 4 %, and in this test, it was at 3.99 %. The formula for the minimum sample size for dichotomous variables (it is a vast majority in the study) is given by equation (Gang 1999):

$$ n = \frac{{Z\left( {p\left( {1 - p} \right)} \right)}}{{d^{ 2} }} $$

where *n*—sample size; Z—statistics for the desired level of confidence; *p*—an estimate of the expected proportion of the variable of interest in the population; *d*—half the width of the desired interval.

The sample size n is 603 patients. For a 95 % confidence level, from a standard normal distribution, *Z* = 1.96 (Domański 2001). The value *d* is at the level of 3.99 %, assuming that the position of the variables of interest is approaching unity (assumed *p* = 99 %).

The following issues regarding patients benefiting from the aesthetic treatments have been examined:

- age distribution, taking gender into account;
- evaluation of the factors essential to a happy life, including the influence of God and God’s Providence on patients’ happiness;
- evaluation of the quality of life of patients benefiting from the aesthetic medicine treatments (cosmetic medicine);
- causal attribution style, that is, who or what did the successes or failures in the past year depend on.

## Results

- Age distribution of people benefiting from aesthetic medicine treatments, taking gender into account.

**Fig. 1:**
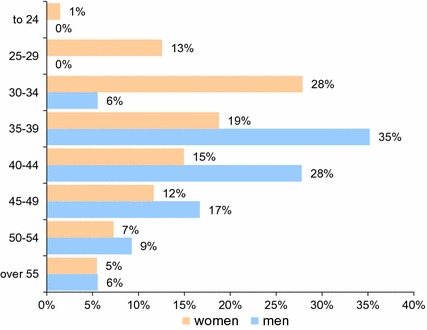
Age distribution of people benefiting from aesthetic medicine treatments, taking gender into account; women: *n* = 549, men *n* = 54

The average age of patients benefiting from the aesthetic medicine treatments (cosmetic medicine) in Poland is 38 years; most often, they are people from 30 to 49 years of age (74 %). Women significantly earlier begin to use such treatments. In a group of men, the greatest interest in the use of aesthetic medicine treatments begins before 40 years of age (which is probably associated with the midlife crisis); however, from 45 years, it steadily decreases. Ageing women much less frequently resign from the treatments. Nevertheless, men and women over 55 years of age significantly lose interest in the aesthetic medicine treatments (Fig. 1).

- Evaluation of factors essential to a happy life of patients benefiting from aesthetic medicine treatments in Poland.

**Fig. 2:**
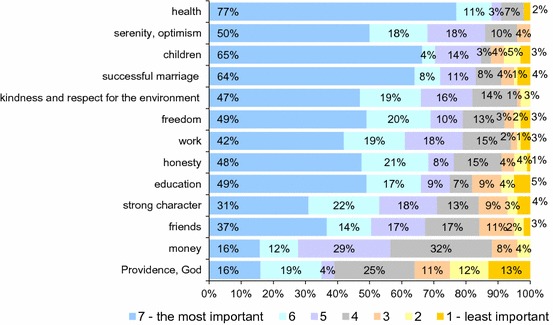
What do you consider the most important condition for a successful, happy life? *n* = 603

People perceive happiness in many different ways. For some, the condition for happiness will be owning a lot of money, for others enjoying a good health, and for others, family and a successful marriage or appropriate education and work. Patients were asked to classify, in the order of importance (1—the least important, 7—the most important), the individual factors that could bring a sense of happiness (Fig. 2).

For patients benefiting from the aesthetic medicine treatments (cosmetic medicine), the most important prerequisite for happiness is health (91 %). The second is cheerfulness and optimism (86 %). Family; children rank also very high (84 %), followed by successful marriage (83 %). The least important condition for happiness is the Providence of God that appears in 36 % of evaluations in the lower half of the scale. The next is education (18 %), a strong character (16 %) and friends (15 %).

- Evaluation of the quality of life of patients benefiting from the aesthetic medicine treatments.

**Fig. 3:**
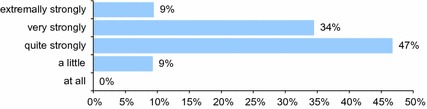
How much do you enjoy life? *n* = 603

Evaluation of the “level of happiness” and joy of life is virtually not possible other than the subjective evaluation and declaration. Therefore, respondents were asked how much they enjoy their lives (Fig. 3).

It is easy to notice that all aesthetic medicine patients enjoy life to some extent. The vast majority of them say that they really enjoy their life (91 %).

- Causal attribution style.

The causal attribution scale used in the study is supposed to provide answers to the question: who or what do the aesthetic medicine patients make responsible for the quality of their own lives: themselves, the authorities, friends, strangers, fate/Providence of God or aesthetic medicine treatments (cosmetic medicine) (Table 1).

**Table 1:** On whom or what did the successes or failures in the past year depend on?

Whom or what did the past year depend on?	“Social Diagnosis”	Aesthetic medicine (cosmetic medicine) patients (%)
The authorities	7.5 %	34
Myself	70 %	86
Aesthetic medicine treatments (cosmetic medicine)	n/a	56
Fate (Providence of God)	41 %	27
Friends	n/a	47
Other people	26 %	21

*n/a* not applicable *n* = 603

**Fig. 4:**
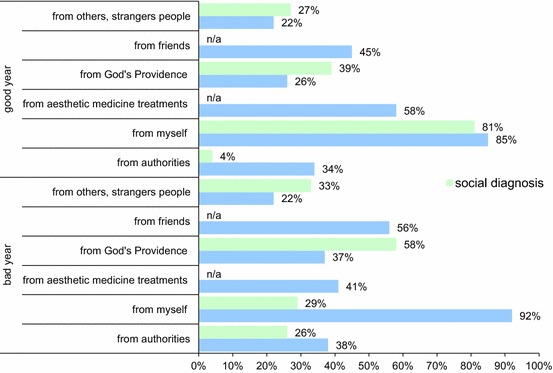
Comparison of causal attribution style “of the average Pole” (Czapliński and Panek 2009) and the aesthetic medicine patient in the separation whether the past year was successful or not (*blue colour*—patients of aesthetic medicine, *n/a* not applicable). *n* = 603

Aesthetic medicine patients more often (16 % p.p.) see their impact on whether the past year was successful or not, and 4.5 times more often indicate a significant impact of the authorities on their life than the average Pole does. In a much lower degree (14 % p.p.) than average Poles, aesthetic medicine patients recognize the impact of fate and Divine Providence on the past year, similar to the impact of other people (5 % less p.p.) (Fig. 4).

First of all, it is worth noting that all patients using aesthetic medicine treatments (cosmetic medicine) very highly evaluated their own impact on whether last year was successful or not. Even in the case of an unsuccessful year, assessment of one’s own impact on this situation has increased. Strangers have had a limited and permanent influence on the aesthetic medicine patients which does not change in respect of whether the year was successful or not. In case of “the average Pole ”(Czapliński and Panek 2009), it looks different, the influence of strangers on the past year has been indicated, and their impact increases in the situations where the last year was unsuccessful. However, patients benefiting from aesthetic medicine treatments, in case of unsuccessful year, are more likely to blame their friends for it. Unfortunately, the study lacks data from “Social Diagnosis” (Czapliński and Panek 2009). When it comes to fate and God’s Providence then again, aesthetic medicine patients much less often point out that this factor is an indicator of the past year’s success or failure comparing to the average Poles, but in both cases, the frequency of the indications on the fate and Providence of God as the cause increased if the past year was unsuccessful.

Aesthetic medicine patients are much more aware of the impact of the authorities on whether their year was successful or not than the average Pole. Also, in case of an unsuccessful year, the tendency to indicate the authorities as the reasons for such state does not increase so dramatically as in case of the average Pole. In general, it is possible to notice an increased desire to indicate the individual factors that could, in subjects’ opinion, have an impact on whether the past year was successful or not. But there is one exception. A lot of patients are more likely to point to the aesthetic medicine treatments (cosmetic medicine) as one of the reasons that the past year was successful.

## Discussion

The study carried out by psychologists has shown that people who consider themselves as attractive rate higher their mental health and are more extraverted and happy (Feingold 1992). Their quality of life is significantly improved. Langlois in his research notes similarly that the attractive people, in accordance with the stereotype “beautiful is good”, are considered to be (in addition to greater intelligence, more assertiveness, more confidence) happier than the average ones (Langlois et al. 2000). This is related to the fact that beautiful people, since birth experience more smile, are milder judged by environment and a greater capacity is seen in them. Such person becomes more confident and enjoys the world and the environment (Cabrić and Pokrywka 2010). Aesthetic medicine patients are also satisfied and happy people.

In this work, all the aesthetic medicine patients enjoy the life to some extent; none of the interviewed replied that they do not enjoy the life. Up to 91 % of them say that they highly enjoy it. This percentage is significant, compared to other social groups, for instance, to patients in the perimenopausal period. Here, Polish women evaluate their quality of life as good in 41 %, while Greek women only in 17.6 % (Krajewska 2007). It is definitely better among American women, as it has been shown that 51 % of them during menopause and after menopause are very satisfied and experience the happiest period in their life (Ojeda 2008); however, patients after aesthetic medicine treatments (cosmetic medicine) evaluate their quality of life the highest.

For patients benefiting from the aesthetic medicine treatments (cosmetic medicine), the most important prerequisite for happiness is their health (91 %). The second is cheerfulness and optimism (86 %). Family; children rank also very high (84 %) and successful marriage (83 %). The least important condition of happiness is the Providence of God that appears in 36 % of evaluations in the lower half of the scale. The next is education (18 %), a strong character (16 %) and friends (15 %).

The style of the causal attribution is a human tendency to search for reasons for one’s own state of behaviour and effects of the actions or state of behaviours and the effects of other people’s actions in certain factors (Czapliński and Panek 2009). The causal attribution scale used in the study provided an answer to the question whether the past year has been successful or not and who or what do the aesthetic medicine patients make responsible for the quality of their own lives: themselves, the authorities, friends, strangers, fate/Providence of God or aesthetic medicine treatments. In many studies, this question is related to a confirmed attributive inclination in the service of ego (“whatever good—it is me, whatever wrong—it’s not me”) and the theory of social ingratitude, which says that the social reception of changes at the macro-level is asymmetrical: those who from the beginning are gaining on these changes show little appreciation to their creators, they mainly look for causes of improvement of their own life in themselves, and the change for better, they experience quite poorly. Those who claim to be harmed as a result of the implementation of reforms blame the authors of the reforms for the deterioration of the conditions of their life and experience changes for worse much stronger (Czapliński and Panek 2009, Czapliński 2000, 2001). Therefore, who the responsibility is assigned to depends on the direction of the perceived change in the quality of one’s own life.

It is noticed that in comparison with the results of the “Social Diagnosis” (Czapliński and Panek 2009), which has an impact on the entire Polish population, the results obtained in the study of aesthetic medicine patients considerably differ from them. Aesthetic medicine patients can more often (about 16 p.p.) see their impact on whether the past year was successful or not. This is probably the greater awareness and social status of patients benefiting from such treatments, because the average aesthetic medicine and anti-ageing patient in Poland is a young woman, well established, educated and happy with the life. This can be seen especially in the impact of authorities on the past year—aesthetic medicine patients 4.5 times more often show a significant impact of the authority on their life than does the average Pole, because the average Pole much more frequently blame fate, Providence and other people for deterioration in the quality of his life, while not recognizing the impact of the authority on such situation. Even if the year was relatively quiet a calm year in the politics, the aesthetic medicine patients, as a better-situated social group, can detect not only simple political events, but also the less noticeable to the average Pole, such as, tax system affecting the economy. One can notice that patients benefiting from aesthetic medicine treatments (cosmetic medicine) are much more aware and feel more responsible for their fate than the average Pole.

Aesthetic medicine patients differ from the average people; this is indicated by the causal attribution style, that is, who will the responsibility for the specific changes in the quality of life be assigned to. In addition, over 90 % of the population declare that is deeply devout and 41.6 % of adults are reported to systematically participate in church services and other religious ceremonies (Czapliński and Panek 2013). In such case, the response in this study should be different, because of the deep faith, but only 36 % of patients responded that their happiness depends on God and Providence. In addition, according to the “Social Diagnosis” (Czapliński and Panek 2013), the impact of religious practices on the mental well-being of Poles deteriorates yearly that would explain the low results in this work. Most likely, such data result from the fact that patients strongly tend to think about God, in the toughest moments of their lives, when they are faced with a disease (oncology, surgery, etc.), while the aesthetic medicine is the only field of medicine that deals with a healthy patient. Patients benefit from treatments to improve their appearance, in the aesthetic sense. Keep in mind that a typical aesthetic medicine patient in Poland is a young woman, well established and educated, while most of the religious groups in Poland include: women above the age of 60, primarily an inhabitant of the village, with basic education, mostly annuitants and retired, who rarely or never use aesthetic medicine from the financial reasons and their beliefs (Czapliński and Panek 2013).

Therefore, patients after aesthetic medicine treatments (cosmetic medicine) in Poland are a group of people who are highly assessing their quality of life and who are happy; however, to a small extent, they attribute these reasons to God or Divine Providence, even in such a Catholic country as Poland.

## Conclusions

- The average age of patients benefiting from the aesthetic medicine treatments (cosmetic medicine) in Poland is 38 years;
- 91 % of patients benefiting from the aesthetic medicine treatments (cosmetic medicine) claim that they strongly enjoy life;
- For patients using the aesthetic medicine treatments (cosmetic medicine) in Poland, God and Divine Providence (36 %) and education (18 %), a strong character (16 %) and friends (15 %) are the least important conditions for happiness. The most important prerequisite for happiness for them is health (91 %), cheerfulness and optimism (86 %) and family; children (84 %) and successful marriage (83 %);
- Aesthetic medicine patients much less (about 14 % p.p.) than the average Poles tended to be influenced by fate and Providence of God in the past year;
- Aesthetic medicine patients more often (about 16 % p.p.) notice their own impact on whether the past year was successful or not, and 4.5 times more often indicate a significant impact of the authority on their life than does the average Pole.
